# Supplementary figures and images for: Integration of mRNA-miRNA Reveals the Possible Role of PyCYCD3 in Increasing Branches Through Bud-Notching in Pear (Pyrus bretschneideri Rehd.)
Source: Plants (Basel). 2024 Oct 18;13(20):2928. doi: 10.3390/plants13202928 (PMC11511176; doi:10.3390/plants13202928)

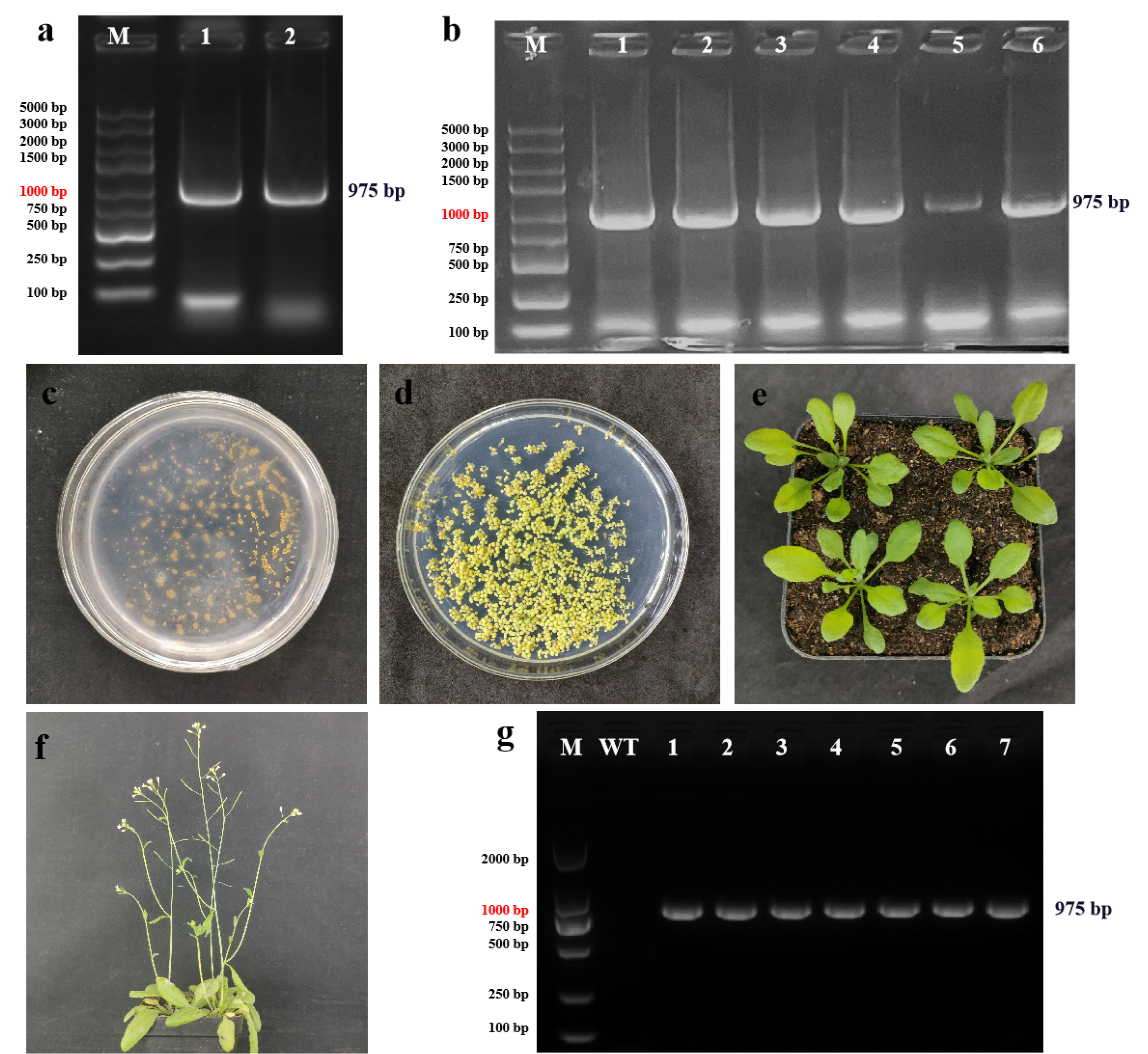

Supplement: Supplementary file 1 [file plants-13-02928-s001.zip › Figure S1.png]
